# Environmentally Friendly Melt-Processed Chitosan/Starch Composites Modified with PVA and Lignin

**DOI:** 10.3390/polym13162685

**Published:** 2021-08-11

**Authors:** Weronika Janik, Anna Wojtala, Anna Pietruszka, Gabriela Dudek, Ewa Sabura

**Affiliations:** 1 Łukasiewicz Research Network-The Institute of Heavy Organic Synthesis “Blachownia”, Energetyków 9, 47-225 Kędzierzyn-Koźle, Poland; anna.wojtala@icso.lukasiewicz.gov.pl (A.W.); anna.pietruszka@icso.lukasiewicz.gov.pl (A.P.); ewa.sabura@icso.lukasiewicz.gov.pl (E.S.); 2 Department of Physical Chemistry and Technology of Polymers, PhD School, Silesian University of Technology, 2a Akademicka Str., 44-100 Gliwice, Poland; 3 Department of Physical Chemistry and Technology of Polymers, Faculty of Chemistry, Silesian University of Technology, Strzody 9, 44-100 Gliwice, Poland; gabriela.maria.dudek@polsl.pl

**Keywords:** chitosan, starch, thermomechanical processing, lignin, PVA

## Abstract

Chitosan/starch-based composites were prepared by thermomechanical processing as an alternative to the traditional solution method, with the aim of fabricating environmentally friendly materials on a larger scale. Different contents and types of lignin and poly(vinyl alcohol), PVA were incorporated into chitosan/starch compositions to improve their mechanical properties. It was demonstrated that the presence of both lignin and PVA increases the values of tensile strength and elongation at break of the composites. Moreover, it was observed that by the selection of a type of lignin and PVA, it was possible to tailor the internal microstructure of the samples. As observed in scanning electron microscope (SEM) micrographs, the introduction of lignin and PVA resulted in the formation of a smooth surface and homogeneous samples.

## 1. Introduction

Due to the increased demand for petroleum materials and the impact of their accumulation in the environment, there is a growing interest in the design of biodegradable and compostable polymers. Among a wide range of biopolymers, chitosan is the second most popular natural polymer. Chitosan is a natural bioactive polysacharide possessing intrinsic antimicrobial activity, exhibiting exceptional physicochemical properties imparted by the presence of a polysaccharide backbone, including biodegradability, biocompatibility, and low toxicity. Due to its properties, chitosan has been extensively investigated in many fields, mainly as an environmentally friendly material for packaging applications [1,2,3]. Moreover, the good film-forming properties of chitosan allows the production of different films and coating materials [2,3,4,5]. Chitosan is obtained by a partial deacetylation of chitin, and the degree of deacetylation is proportional to the transformational degree of chitosan from chitin [1]. Most chitosans are insoluble in water and in commonly used organic solvents, but they can be dissolved in acidic aqueous solutions of pH below 6.3 [5]. Still, the use of chitosan at large scale is limited. The reason for this is its high costs and worse properties when compared to traditional petroleum-based plastics, such as lower barrier against water vapor, and thermal and mechanical strength. Fortunately, these properties can be improved by blending chitosan with other biopolymers [4,5].

Chitosan-based films and coatings have been recently intensively studied [2,3,4,5,6,7,8,9,10,11,12,13,14,15,16,17,18,19,20,21,22,23]. Most of the research has been focused on evaluating different preparation conditions (including the processing method) and incorporation of additives to improve the properties of composites. Currently, there are two main methods to produce films from chitosan: a wet method based on solvent casting, and a dry method [6], with the preference for wet method due to its unquestionable simplicity [2,3,4,5,6,7,8,9,10,11,12,13,14,15]. The obtained materials, however, are fragile at small thickness. Additionally, solvent casting is hard to scale up from laboratory to industrial level, because it is a long-lasting process [6]. Only a few literature reports describe the method of chitosan plasticization by one-step extrusion process, but in these composites the amount of chitosan is less than 10 wt.%.

Recently, blends of chitosan/PVA, chitosan/starch, or chitosan/lignin have been greatly studied as promising materials for industrial applications [7,8,9,10,11,12,13,14,15,16,17,18]. Polymer blends of chitosan and PVA exhibited good thermal stability, unique morphology and reduced solubility in acid solution. FTIR study showed that PVA and chitosan were considered miscible and compatible owing to hydrogen-bonding interaction [7,8]. Moreover, the use of sorbitol as a plasticizer caused an increase in the elongation at break [9]. Composites of chitosan and starch showed good mechanical properties, especially after the addition of more starch content [10]. Furthermore, the addition of keratin fibers increased the glass temperature and the storage modulus of the obtained films [11]. The other type of biocomposite film that has recently been a subject of investigations is chitosan/lignin [12,13,14,15]. The FTIR data confirmed the formation of hydrogen bonding between the hydroxyl and carbonyl groups in chitosan with carbonyl, hydroxyl, and ether groups in lignin [12,15]. Additionally, a salt was formed between the protonated amino group of chitosan and the carboxylic group of lignin. The bond formation between the components led to the formation of a crosslinked elastic framework carrying the main mechanical load. Introduction of lignin resulted in an insignificant decrease in tensile strength and modulus of the obtained composite films, while it greatly decreased the cost of formed films.

Polymer blending by mixing two natural biopolymers (chitosan and starch) with PVA was investigated in the following studies [16,17,18]. The infrared spectra confirmed the good compatibility of PVA with chitosan/starch composite [16]. Additionally, several beneficial effects of PVA addition to the composite were observed: an increase of film flexibility and elongation at break [16], pore disappearance and smoothing of the surface [17], and improved mechanical and functional properties without the loss of biodegradability [18].

Another method for preparing polymer films and coatings is thermomechanical processing. As opposed to the casting method, this method can be used in industrial conditions, which allows the preparation of biodegradable materials on a larger scale. However, the plasticization of chitosan is necessary before applying this method. Glycerol, sorbitol, and xylitol are frequently used as plasticizers, and their presence provides thermoplastic material with good mechanical properties [19,20,21,22]. Furthermore, thermomechanical processing allows the use of the conventional tools for polymer processing, such as extruders, kneaders, injection molding, etc., enabling the production of a wide variety of forms and shapes in contrast to a wet method, in which only casted films are produced.

More recently, chitosan has been plasticized during a one-step extrusion process [23,24,25,26]. The plasticizing system (glycerol with acetic acid) was mixed with polyethylene [21] or keratin fiber [25]. Extrusion compounding was shown to be a promising method for the industrial processing of chitosan/starch composites due to the possibility of further molding of the materials and its high productivity. It could be used to fabricate a material with a high dispersion and distribution degree of the chitosan phase in starch.

Taking into account the versatility of thermomechanical processing, we used this method to fabricate chitosan/starch composites with lignin and PVA. To the best of our knowledge, similar composites have been only obtained by conventional casting methods [16,17,18]. Moreover, every previous work describing thermomechanical-processed chitosan [19,20] was associated with its low content in the composite. In our case, chitosan was one of the main components of the composite, with a content of about 30 wt.%. We measured the hydrophilic, mechanical, and thermal properties of investigated samples and analyzed their variation with sample composition.

## 2. Materials and Methods

### 2.1. Materials

Chitosan 30–100 cps (M_W_ = 250,000, DD ≥ 90%) and chitosan 100–300 cps (M_W_ = 890,000, DD ≥ 90%) were purchased from Glentham Life Sciences Ltd. (Corsham, UK).. Each composite contained native potato starch purchased from Przedsiębiorstwo Przemysłu Ziemniaczanego “Trzemeszno” Sp. z o. o. (Trzemeszno, Poland), glycerol from Eurochem BGD Sp. z o.o. (Tarnów, Poland) and acetic acid 20% solution. Three types of lignin were used (dealkaline L0045 from Tokyo Chemical Industry (Tokyo, Japan), and Kraft (sulfate) ones: Lineo Classic and Lineo Classic W from Stora Enso (Stockholm, Sweden)). Three different PVA were used: Elvanol 71–30 (viscosity 27.0–33.0 mPa.s, Mw ~ 100,000, fully hydrolyzed) was provided by Kuraray Europe GmbH (Hattersheim am Main, Germany), Mowiol 20–98 (viscosity 18.5–21.5 mPa.s, Mw ~ 125,000, 98.0–98.8 mol% hydrolysis) purchased from Merck (Darmstadt, Germany) and Mowiol 8–88 (viscosity 7–9 mPa.s, Mw ~ 67,000, 86.7–88.7 mol% hydrolysis) were also purchased from Merck (Darmstadt, Germany).

### 2.2. Sample Preparation

The formulation of chitosan/starch-based samples is shown in Table 1. The appropriate amount of glycerol was incorporated in the chitosan powder and manually mixed. Then, an adequate amount of acetic acid aqueous solution (20 wt.%) was added to the chitosan/glycerol mixture to obtain a paste with final chitosan concentration of 30 wt.%. Then, starch, lignin, and PVA were introduced. The mixtures were then mechanically blended in a HaakePoly-Lab QC Reomix 600 internal batch mixer at 80 °C for 4 min, with a rotor speed of 40 rpm. Finally, the resulting materials were first hot-pressed at 110 °C, 250 bar pressure for 7 min and immediately cooled at room temperature, in the press, for 2 min.

### 2.3. Moisture Content (MC), Swelling Degree (SD), and Total Soluble Matter (TSM)

Moisture content (MC) was determined by a gravimetric method. Weighed samples of a mass ~1 g were dried in an air-circulating oven at 105 °C for 24 h. After this time, the samples were once again weighed. Moisture content was calculated as the percentage of weight loss based on the original weight. Triplicate measurements of MC were conducted for each sample, and the average MC was calculated. A swelling degree (SD) study was performed by putting dried samples into 100 mL distilled water at room temperature for 24 h. All samples were weighed before and after swelling. Total soluble matter (TSM) was determined by immersing samples in 100 mL of distilled water at 25 °C for 24 h. After this time, the samples were dried in an air-circulating oven at 105 °C for 24 h. TSM was calculated in relation to the dry mass, and it was expressed as the percentage of sample dry matter solubilized.

### 2.4. Mechanical Properties

Tensile tests were performed by means of an Instron 4466 machine. The tests were carried out at ambient temperature with the indications of PN-EN ISO 527: 2012, sheets 1 and 2. A constant stretching rate of 100 mm/min was applied. Mechanical properties were determined from the average of five measurements.

### 2.5. Scanning Electron Microscopy (SEM)

Surface morphology of the composite samples was studied using a HITACHI TM3000 scanning electron microscope at a 10 kV accelerating voltage and a chamber pressure of about 9 × 10^−3^ Pa. Cross-sectional SEM samples were made by freeze fracturing with liquid nitrogen.

### 2.6. Thermogravimetric Analysis (TGA)

Thermogravimetric analysis was carried out using a Mettler Toledo TGA 2 Thermobalance. The samples (5 mg) were heated up in an open platinum crucible (Pt 70 μL), in the temperature range 30–1100 °C, with the heating rate β = 20 °C/min, in the dynamic (100 mL/min) nitrogen or air atmosphere. The thermographs were analyzed with the use of the STARe Thermal Analysis Software.

### 2.7. Differential Scanning Calorimetry (DSC)

DSC measurements were performed using a Mettler Toledo DSC 822e Differential Scanning Calorimeter. Measurements were carried out in two or three stages for m_S_ ~ 5 mg in an aluminum crucible (Al 40 µL) closed with perforated lid, under a nitrogen flow of 50 mL/min according to the temperature program below:

The investigated samples were first heated from 0 °C to 220 °C with the heating rate β = 10 °C/min, then cooled to 0 °C at 10 °C/min, and finally, the second heating was performed to 220 °C at 10 °C/min. The investigated samples were first heated from −60 °C to 220 °C at 10 °C/min, then from −60 °C to 300 °C with the same heating rate.

## 3. Results

### 3.1. Moisture Content, Swelling Degree, and Total Soluble Matter

**Figure 1:**
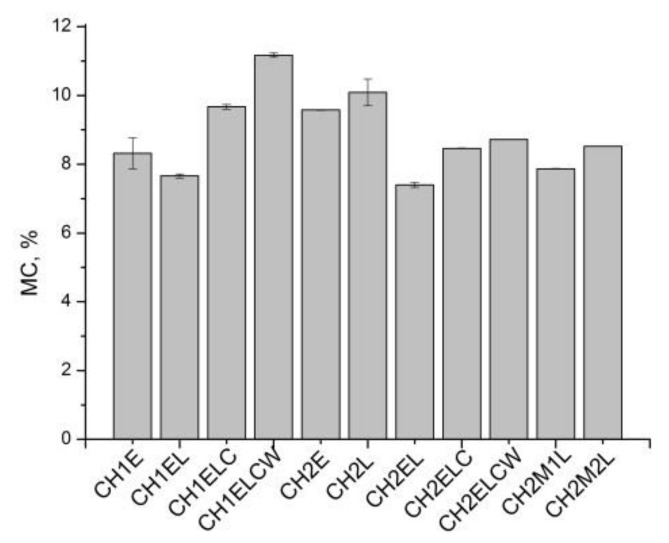
Moisture content (MC) for chitosan/starch-based samples.

**Figure 2:**
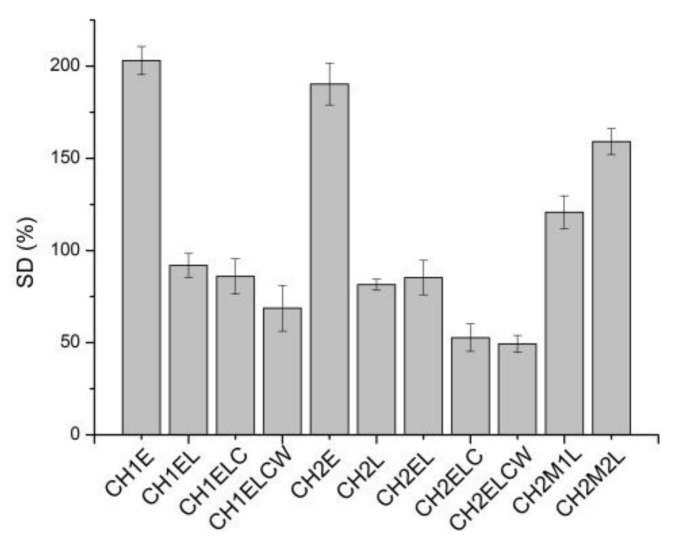
Swelling degree (SD) for chitosan/starch-based samples.

**Figure 3:**
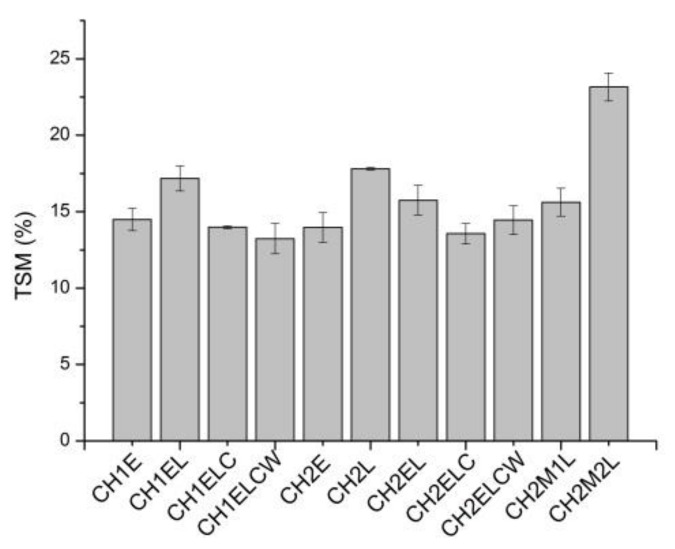
Total soluble matter (TSM) for chitosan/starch-based samples.

Figure 1, Figure 2, Figure 3 show the moisture content (MC), swelling degree (SD), and total soluble matter (TSM) of the chitosan/starch-based samples. As can be seen in Figure 1, MC values are similar (around 10%) for all investigated samples. Regarding the composites prepared with different chitosan (CH1 and CH2), it is noticed that the type of chitosan has a slight influence on the moisture content, of about 1 wt.%. In the case of a swelling degree (Figure 2), it can be noticed that the highest value of this parameter (about 200%) is obtained for compositions CH1E and CH2E, with the absence of lignin. This behavior can be explained by the physical crosslinking of hydrophilic polymers with lignin via H-bonds. Lignin partially “blocks” hydrophilic sites of glycerol and chitosan by hydrogen bonds with OH groups, leaving less OH groups to interact with water molecules, resulting in lower SD values [27]. Significant values are also observed for CH2M1L and CH2M2L samples, both containing PVA, which is not fully hydrolyzed. PVA is widely recognized as a crystalline water-soluble polymer. The degree of crystallinity depends on the degree of hydrolysis, and higher degree of hydrolysis causes higher crystallinity. According to [28] the swelling behavior closely correlates with the crystallinity. Therefore, the increase in the swelling ratio is expected to arise from the degradation of crystalline phase leading to the increase in the presence of amorphous one. The lowest SD value is observed in the case of CH2ELC and CH2ELCW samples where kraft lignin is used. Among the various types of lignin, kraft lignin is much less soluble in water at neutral or acidic pH because of the lack of hydrophilic groups [29]. Considering the total soluble matter for chitosan/starch-based samples, it can be noticed that this parameter is similar for all investigated samples. The exception is the CH2M2L sample, where TSM reaches a value 30% higher than for other samples. Compared to other samples, only this sample contains PVA with the lowest percentage of hydrolysis (86.7–88.7 mol%). The partially hydrolyzed PVA possesses residual acetate groups, which are essentially hydrophobic, and can generate a steric hindrance that disturbs the arrangement of the intermolecular chains in PVA and inhibits the formation of hydrogen bonds between the molecular chains. The presence of an adequate number of these acetate groups enhances composite solubility in water.

**Figure 4:**
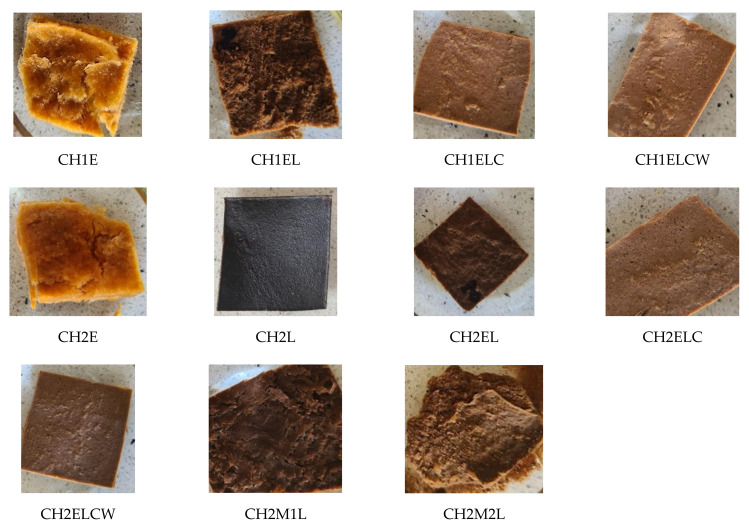
Chitosan/starch-based samples after immersion in water for 24 h.

Figure 4 shows optical images of samples after immersion in water for 24 h. As can be seen, chitosan/starch-based samples swell after immersion in water. The smoothest surface after swelling is observed for the sample CH2L suggesting its most homogeneous structure. Nevertheless, it can be seen that most of the samples retained their structure and shape. The most visible degradation of a sample can be observed for CH2M2L, which suggests its lowest resistance to water, as confirmed by the results of SD and TSM measurements. However, it can be seen that swelling is lower for samples with lignin and regarding to the obtained results, the lowest value is observed for the composite modified with LCW. This indicates that the type of lignin influences on the structure of the investigated composites, probably due to the presence of chemical interactions between amino groups in chitosan and methoxy together with hydroxy groups in lignin [30]. A tighter structure leads to a decrease in swelling.

### 3.2. Mechanical Properties

**Figure 5:**
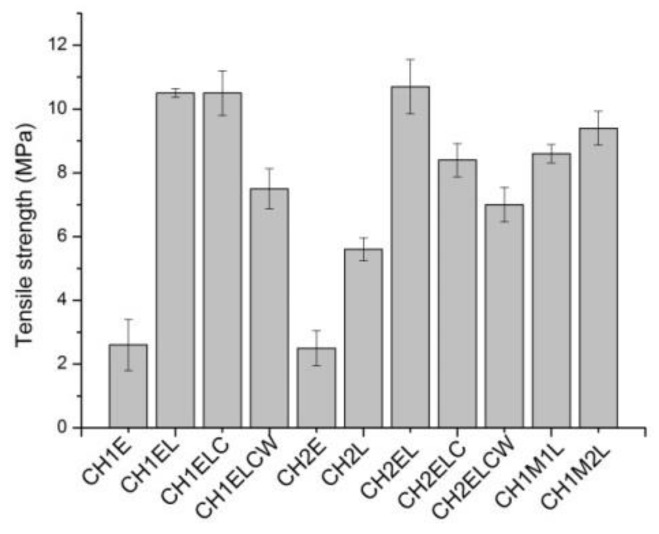
Tensile strength for chitosan/starch-based samples.

**Figure 6:**
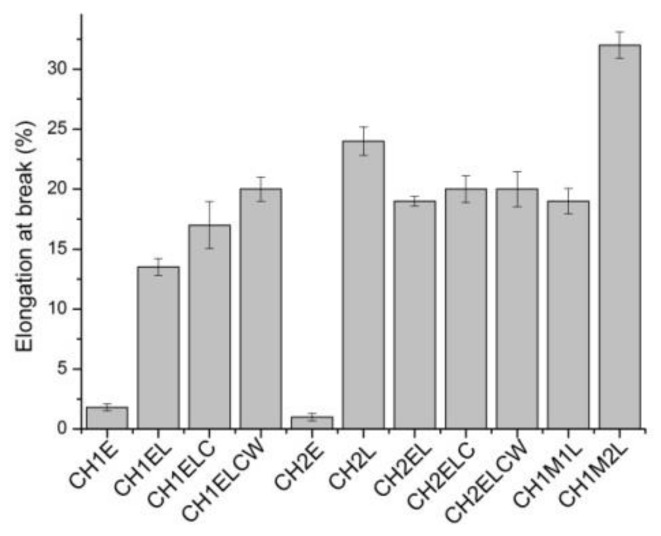
Elongation at break for chitosan/starch-based samples.

**Figure 7:**
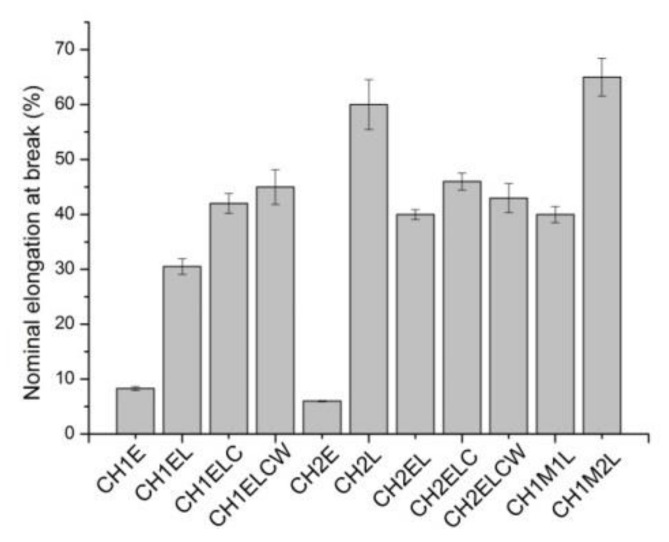
Nominal elongation at break for chitosan/starch-based samples.

The effect of lignin and PVA content on the mechanical properties is shown in Figure 5, Figure 6, Figure 7. Regarding the composites prepared with different chitosan (CH1 and CH2), it is noticed that the type of chitosan has a slight influence on the mechanical properties of composites. In the case of tensile strength, a difference of about 2 wt.% is observed whereas elongation at break and nominal elongation at break vary by about 4% for both types of chitosan. However, tensile strength and elongation at break increases for samples modified with lignin, which is expected to arise from the interactions between chitosan/starch and lignin. This result is different from described by Rosova et al. [15], where the addition of lignin resulted in a decrease in tensile strength and elongation at break in chitosan samples. Even though mechanical properties of the samples can be mainly linked to the physicochemical characteristic of the polymers and plasticizers, it should be also taken into account that interactions between polymers and plasticizer could vary with the inclusion of additional ingredients, such as lignin or PVA. Different results were observed by Chen et al. [12], where tensile strength increased with the increase in lignin content up to 20 wt.% and decreased after reaching this value. Meanwhile, the value of elongation at break also increased, so it was concluded that lignin can be used as a compatibilizer in chitosan-based samples. Regarding the composites prepared with different lignin (L, LC and LCW), it is noticed that the type of lignin has an influence on the mechanical properties of composites. In the case of tensile strength, the highest value is observed for CH1EL and CH2EL, where lignin L is used. Considering elongation at break and nominal elongation at break, a slight difference is noted, about 3 wt.%. It is noticed that PVA also influences the mechanical properties of composites. Samples modified with PVA exhibit an increase in tensile strength and decrease in elongation at break. The differences in the results between the types of PVA are associated with distribution and density of intermolecular and intramolecular interactions in the network created in chitosan/starch samples modified with PVA [31]. Regarding the composites prepared with different PVA (E, M1 and M2), the highest value is observed in the case of the CH2M2L sample, which contains PVA with the lowest percentage of hydrolysis.

### 3.3. Morphology

**Figure 8:**
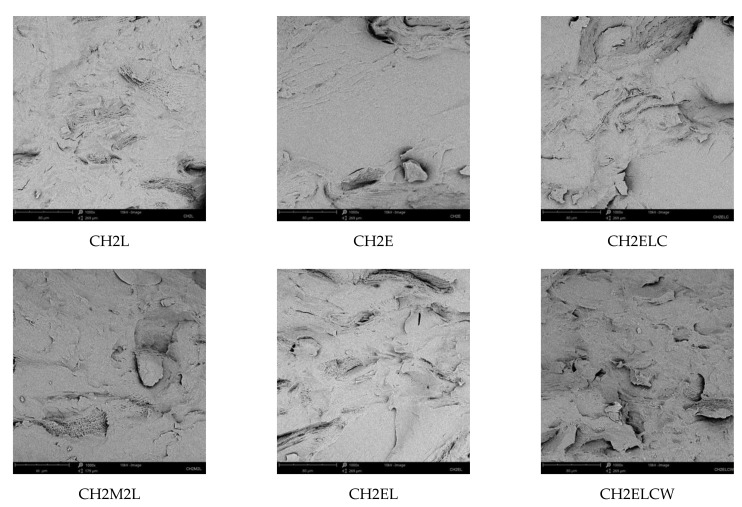
SEM images of chitosan/starch-based samples modified with PVA and lignin, 1000×.

Figure 8 shows scanning electron microscopy (SEM) images of chitosan/starch-based samples. For all samples, a homogeneous structure is observed demonstrating that the chitosan/starch-based samples modified with lignin and/or PVA had a smooth, flat surface. Lignin is present here in the form of irregularly shaped particles, and PVA seems to be entirely dissolved. SEM images show that regardless of the type of lignin, it is evenly distributed in the polymer matrix, and no voids are noticed in the polymer matrix. The surface of CH2E sample without lignin appears to be relatively smoother than that of CH2L composite (6.5 wt.% L). Despite this, it must be emphasized that proper interfacial interaction is an important factor when lignin or other fillers are to be used to improve mechanical properties [32]. According to the mechanical properties results, it is noticed that lignin significantly increases the mechanical properties of the chitosan/starch-based samples. This indicates good interaction between the components, and it is proven by these SEM images, where the samples showed smooth surfaces. Karua and Saho also prepared starch/chitosan samples with the addition of PVA [17]. In this case, an increasing PVA content ranging from 0 to 10 wt.% resulted in the increase in the tensile strength of blends. Moreover, the corresponding SEM images showed that upon the addition of PVA, the surface of the blend became smooth, and the pores disappeared. In our case, SEM images confirm that the samples with PVA appears to be relatively smoother than those without it. In summary, SEM images confirm the homogeneity of dispersion of lignin and PVA in the chitosan/starch matrix, providing a confirmation that the conditions of processing were appropriate.

### 3.4. Thermal Analysis

**Figure 9:**
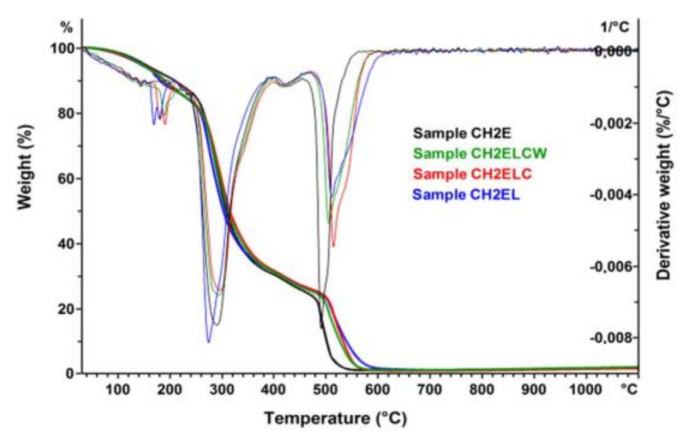
TG and DTG curves of chitosan/starch−based samples.

**Figure 10:**
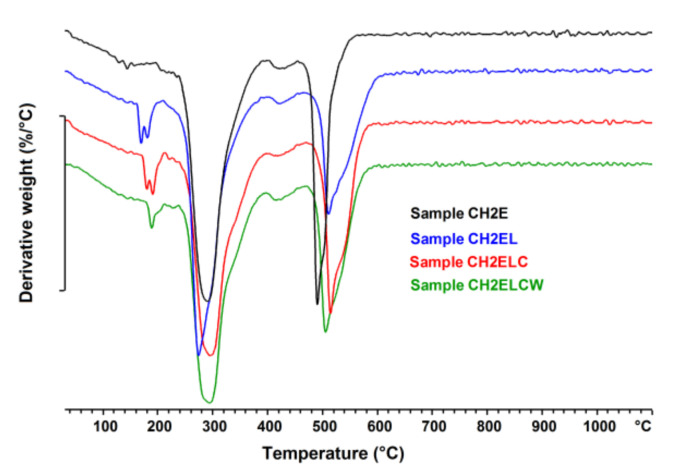
DTG curves of chitosan/starch−based samples.

**Figure 11:**
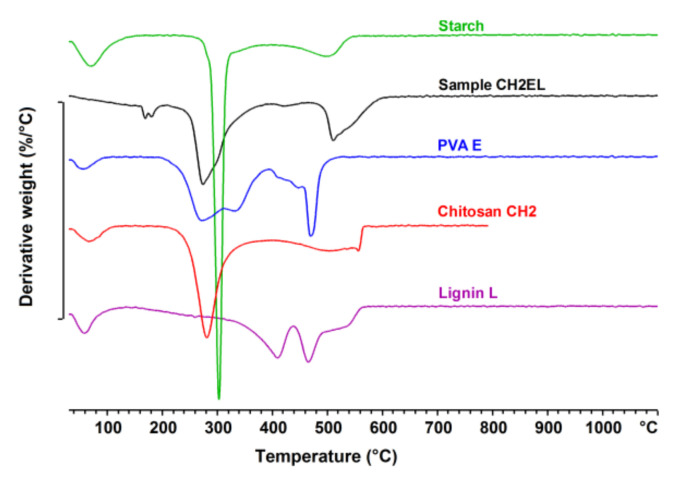
DTG curves of sample CH2EL, pure starch, PVA E, chitosan CH2 and lignin L.

Figure 9, Figure 10, Figure 11 show the TG/DTG curves for the chitosan/starch-based samples as well as pure starch, pure chitosan, pure PVA, and pure lignin. For chitosan/starch-based samples, it can be observed that some specific degradations for chitosan and its crosslinked products occurred. At higher temperatures a degradation of PVA byproducts is also observed [33]. Two main weight losses occurred at the temperature ranges of 200–400 °C and 480–600 °C. The first weight loss is assigned to a rapid decomposition of polymer segments of PVA, chitosan, and starch due to the thermal scission of the polymer backbone. The second weight loss is caused by the degradation of the byproducts generated from PVA during its thermal degradation [34] and lignin. For all chitosan/starch-based samples, the two main weight losses were observed each with one T_onset,_ which indicates significant interactions between the components [35]. It should be also taken into account that the T_onset_ is different for the obtained samples, which should be associated with intramolecular interactions in the samples. The strong hydrogen-bonding interaction between the groups should also increase thermal stability as the formation of a confined structure in the chitosan/starch-based samples [5]. This is observed in the case of samples modified with lignin, where the T_onset_ values in the second stage were higher than for samples without lignin. An additional weight loss in the region of 200 °C can be observed in the DTG curves for samples modified with lignin. This was not observed either on the curve for pure lignin or for the non-modified samples (Figure 11). It should be also noted that for all samples, there is a single event representing the pyrolysis of glycerol observed in the range 130–260 °C [36].

**Figure 12:**
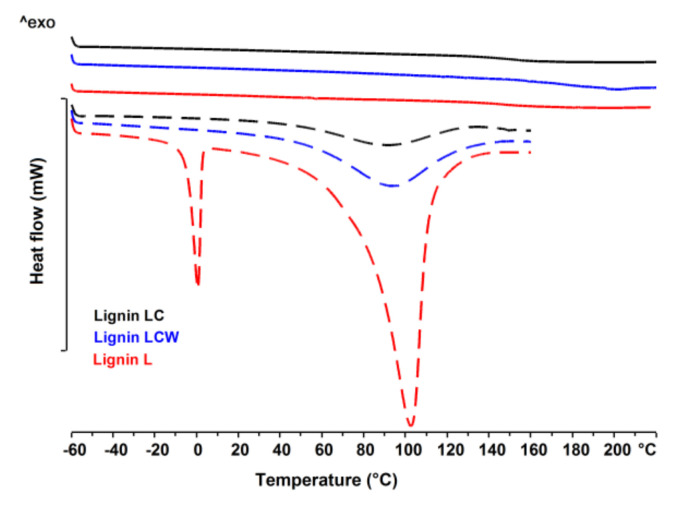
DSC curves of various lignin; 1st heating (dotted line) and 2nd heating (solid line).

**Figure 13:**
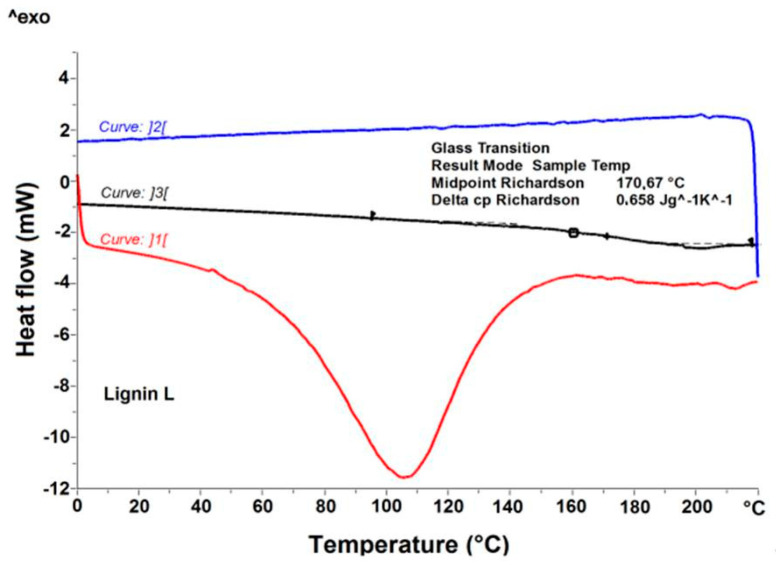
DSC determination of glass transition temperature (T_g_) and change in heat capacity (C_p_) for Lignin L.

**Figure 14:**
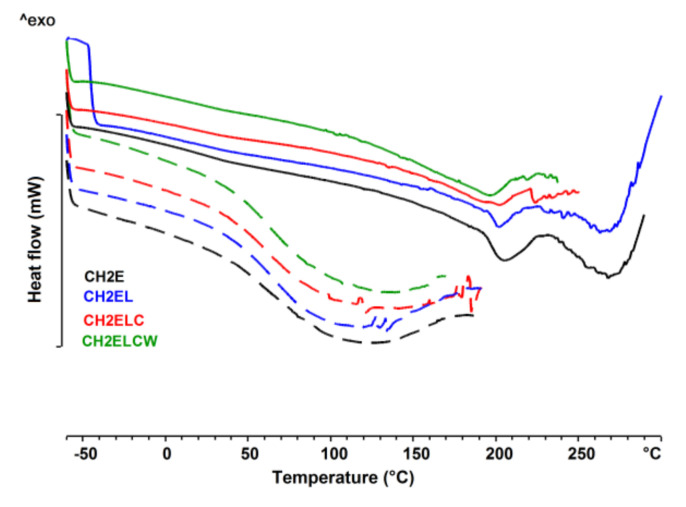
DSC curves of chitosan/starch−based samples; 1st heating (dotted line) and 2nd heating (solid line).

Figure 12 and Figure 13 show the transitions detected in the thermograms of pure lignin used in the chitosan/starch-based samples. The DSC thermogram showed a wide endothermic peak between 20–150 °C assigned to the loss of water. The highest water content is noticed for lignin LCW. Moreover, at temperature of about 0 °C, an additional endothermic peak of the DSC curve is observed, and is associated with the content of freezable water in this type of lignin. Taking into account the enthalpy of fusion and water evaporation of pure LCW, it can be concluded that it contains both freezable water and non-freezable bound water. For both lignin and chitosan, a thermal event was registered. The glass transition temperature was determined using the enthalpy calculation procedure given by Richardson [37], and the values were presented in Table 2. The glass transition temperature is clearly higher for lignin than for chitosan. Moreover, the highest glass transition temperature is observed for lignin L. Figure 14 shows the differential scanning calorimetric scans of the prepared chitosan/starch-based samples. In the first heating curve, a wide endothermic peak between 30–180 °C is observed, which is related to the evaporation of water present in the sample. This transition is no longer observed at the second heating scans. The DSC thermogram shows an endothermic peak between 180–220 °C which is attributed to the melting of PVA, while an exothermic peak starting above 250 °C indicates a thermal decomposition of prepared samples.

## 4. Conclusions

Herein, we describe the fabrication and characterization of environmentally friendly chitosan/starch-based composite materials modified with lignin and PVA. This is the first study reporting samples containing significant amount of chitosan, with a content of about 30 wt.%, prepared via thermomechanical processing. The physicochemical characterization of composites involved the determination of moisture content, swelling degree, total soluble matter, tensile strength, and elongation at break. The results showed that the type of lignin and PVA had a significant influence on the structure of the samples, with the superior mechanical characteristics exhibited by the materials modified with lignin. Furthermore, the addition of PVA was found to increase the tensile strength and decreased the elongation at break. Due to the higher value of tensile strength and similar value of elongation at break, investigated composite materials exhibit better mechanical properties than chitosan/starch compositions produced by extrusion process and containing less than 10 wt.% of chitosan. Moreover, strong hydrogen-bonding interaction between the groups in the chitosan/starch-based samples increased the thermal stability of investigated composites. SEM images showed a good dispersion of lignin and PVA within the composite material, showing smooth surfaces and a high level of homogeneity. The best results were obtained for samples containing both investigated components, i.e., ~20 wt.% PVA and ~5 wt.% lignin, for which tensile strength and nominal elongation at break were equal to ~9 MPa and ~40%, respectively. The performed study proved that the use of lignin and PVA in chitosan/starch composites is a suitable method for obtaining a thermomechanical-processed material. Due to the fact that thermomechanical processing is the most popular method used in the production of packaging, and biopolymers are popular materials on the packaging market, the presented studies can contribute to broaden the applicability of biopolymers at a large scale. Still, the presented results are preliminary, and further research is necessary to improve material properties for industrial application.

## 5. Patents

The patent was registered under the number P.438269.

## Figures and Tables

**Table 1:** Composition of chitosan/starch-based blends.

Sample	Composition, wt.%					
	Chitosan	Starch	PVA	Lignin	Glycerol	AAAS
CH1E	30.30^CH1^	15.76	21.52^E^	-	10.90	21.52
CH2E	30.30^CH2^	15.76	21.52^E^	-	10.90	21.52
CH1EL	28.74^CH1^	14.94	20.40^E^	5.17^L^	10.35	20.40
CH2EL	28.74^CH2^	14.94	20.40^E^	5.17^L^	10.35	20.40
CH1ELC	28.74^CH1^	14.94	20.40^E^	5.17^LC^	10.35	20.40
CH2ELC	28.74^CH2^	14.94	20.40^E^	5.17^LC^	10.35	20.40
CH1ELCW	28.74^CH1^	14.94	20.40^E^	5.17^LCW^	10.35	20.40
CH2ELCW	28.74^CH2^	14.94	20.40^E^	5.17^LCW^	10.35	20.40
CH2L	36.10^CH2^	18.77	-	6.50^L^	13.00	25.63
CH2M1L	28.74^CH2^	14.94	20.40^M1^	5.17^L^	10.35	20.40
CH2M2L	28.74^CH2^	14.94	20.40^M2^	5.17^L^	10.35	20.40

CH1—Chitosan 100–300 cpi; CH2—Chitosan 30–100 cpi; E—PVA Elvanol 71–30; L—Lignin TCI; LC—Lignin StornaEnso Classic; LCW—Lignin StornaEnso Classic W; M1—PVA Mowiol 20–98; M2—PVA Mowiol 8–88; AAAS—Acetic Acid Aqueous Solution.

**Table 2:** Glass transition temperatures and their associated change in heat capacity.

Sample	T_g_ (°C)	∆C_p_ (J·g^−1^·K^−1^)
Lignin LC	148.4	0.374
Lignin LCW	154.2	0.325
Lignin L	170.7	0.658
Chitosan CH2	89.8	0.457

## Data Availability

Not applicable.
